# Monitoring May 2024 solar and geomagnetic storm using broadband seismometers

**DOI:** 10.1038/s41598-024-81079-6

**Published:** 2024-12-03

**Authors:** J. Díaz

**Affiliations:** GEO3BCN – CSIC, Barcelona, Spain

**Keywords:** May 2024 geomagnetic storm, Seismic instrumentation, Magnetic field variation, Pc5 pulsations, Space weather, Magnetospheric physics, Seismology

## Abstract

The abrupt variations in the magnetic field due to the May 2024 geomagnetic storm have been recorded by broadband seismometers distributed around the world for a time interval of more than 55 h. Signals related to magnetic field variations can be identified in seismic data for frequencies below 10 mHz, but are clearer between 1.5 and 5 mHz, the frequency band corresponding to Pc5 magnetic pulsations. The number of seismological stations that detect these signals varies significantly between the various seismic networks analyzed, but in general they do not exceed 50–60%. The origin of these signals appears to be related to the interaction between telluric currents induced by magnetic field disturbances associated with the geomagnetic storm and the detection systems in broadband sensors, which measure the current intensity necessary to keep the mass of the seismometer fixed. Although detailed calibration of each seismometer will be necessary to use the seismic data to model the waveforms and amplitudes of the magnetic pulsations, the analysis of this data provides a significant densification with respect to the magnetic observatory data, as is of interest to analyze the temporal and regional evolution of the magnetics disturbances associated to the May 2024 solar storm.

**Supplementary Information:**

The online version contains supplementary material available at 10.1038/s41598-024-81079-6.

## Introduction

The May 2024 geomagnetic storm, which occurred from 10 to 13 May 2024 has been classified as a G5-class storm and is considered the most powerful geomagnetic storm since March 1989 ^1^. Associated auroras were observed at many low-latitude sites in both hemispheres, including Spain and Italy in Europe or Texas and Florida in the United States of America, generating great interest in the media and on social networks (e.g. https://www.esa.int/Space_Safety/Space_weather/The_May_2024_solar_storm_your_questions_answered, https://earthobservatory.nasa.gov/images/152815/historic-geomagnetic-storm-dazzles).

Following the arrival of magnetic disturbances generated by large ejections of plasma and magnetic fields from the solar corona during solar storms, the magnetospheric and ionospheric currents undergo large variations, which in turn create secondary magnetic fields, which are systematically recorded by magnetometers. The arrival of these disturbances leads to an abrupt increase in the horizontal component of the intensity of the magnetic field recorded at the Earth’s surface, which is defined as a “Storm Commencement” (SC) and is reported in a list distributed by the International Geomagnetic Indices Service (https://isgi.unistra.fr). In the case of the May 2024 solar storm, the SC was reported to start on May 10 at 17:05 UTC, being unambiguously identified and having a mean amplitude of 69.2 nT. Scientific contributions analyzing different aspects of the May 2024 solar storm are beginning to be published^1–4^.

It is well known that magnetic field variations can affect measurements on broadband seismic instruments, although there is no complete agreement on the mechanism explaining this effect. Most contributions discussing the effect of geomagnetic field perturbations on broadband sensors have focused on considering magnetic field-induced signals as noise that must be removed to improve the analysis of seismic data in the normal mode band (0.3-3 mHz). However, in recent years, several authors have analyzed the potential of using magnetic signals recorded by seismic sensors to improve our knowledge of space weather^5–7^.

This contribution reviews the signals generated by the May 2024 geomagnetic storm on broadband seismic sensors, proving that these data are useful to analyze the temporal and regional evolution of the magnetics disturbances resulting from this exceptional storm. We will first compare magnetic and seismic records and then discuss seismic data acquired at continental and global scales, with a special focus on the seismic data available at high latitudes. The seismic data acquired at local seismic networks and at special sites with co-located seismic and magnetic sensors is analyzed to infer new insights into the mechanism responsible of the broadband seismometers sensitivity of magnetic events.

## The May 2024 solar and geomagnetic storm

**Fig. 1:**
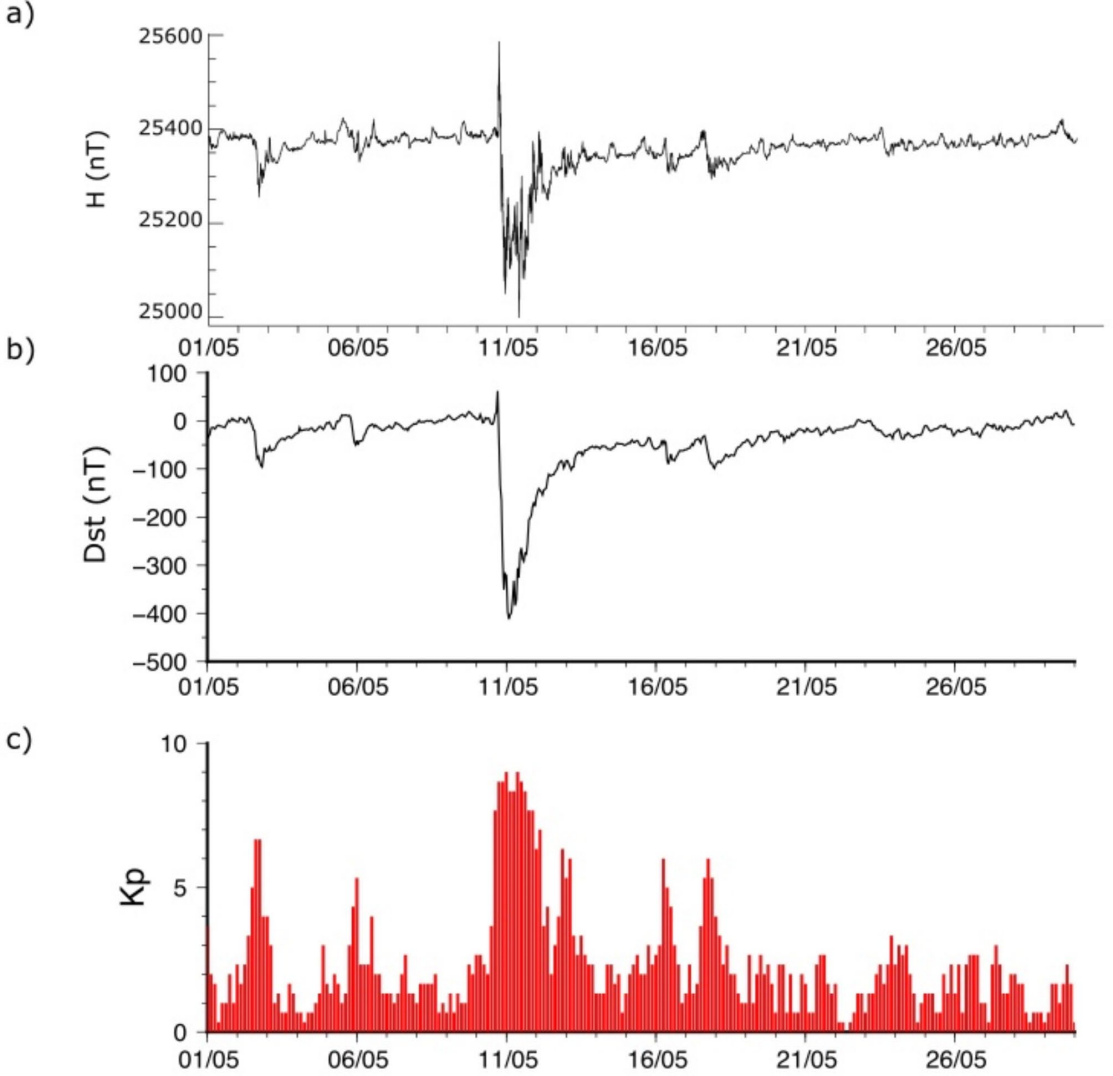
(**a**) Horizontal magnetic intensity (H) recorded at the EBR magnetometer, located in eastern Spain, during May 2024 (**b**) Variation of the Dst index (disturbance in the H at selected sited) during May 2024 (**c**) Variation of the Kp index values during the same period.

The geomagnetic disturbances generated by the May 2024 storm have been recorded worldwide by the magnetometers of the INTERMAGNET network (https://intermagnet.org/). Focusing first on mid-latitude recordings, Fig. 1a shows the horizontal magnetic intensity (H) at the EBR observatory, located in eastern Spain, at a latitude of 40.8ºN. The SC is observed at 17:05 UTC, with an amplitude increase of 110 nT in the first 1.7 min and a maximum increase of 200 nT reached 35 min later. Subsequently, H decreases over a 5-h interval to reach a minimum at 22:38 on 10 May, fluctuating between 25,113 and 25,252 nT for 10 h 40 min then abruptly decreases to a minimum value of 25,000 nT at 9:48 UTC on 11 May, nearly 17 h after storm onset. Thereafter, H recovers more or less steadily to reach pre-storm values in the early hours of 20 May. Similar observations have been reported by Spogli et al.^3^, who analyzed satellite and ground-based data over Italy and reported a SC with a 3-min rise time and amplitudes ranging from 100 to 139 nT, with H increasing over an interval close to 30 min after the SC. Using data from the SER observatory in a low-latitude region of Chile (29.827ºS), Lazzús and Salfate^2^ divided the storm into two phases; a main phase beginning on May 10 at 17:05 and lasting 8 h and a recovery phase beginning in the early hours of May 11 and extending for several days. Supplementary Fig. S1 shows examples of magnetograms acquired at magnetic stations located along a north-south profile, evidencing the large differences in signals as a function of latitude.

To avoid latitudinal differences, magnetic disturbances are monitored on a global scale using indices such as Dst or Kp. The Dst index (https://wdc.kugi.kyoto-u.ac.jp/dstdir/dst2/onDstindex.html) measures the disturbance of the horizontal component of magnetic intensity (H) using data from four magnetic observatories located between 40º N and 40º S and providing an hourly measurement. According to this index, the storm began with a sudden increase of 44 nt followed by a rapid decrease until reaching an extreme value of -412 nT on May 11 at 02:00 UTC (Fig. 1b). The Dst index gradually recovered to pre-storm values, but its values remained around − 100 nT until May 12 at 17:00 UTC.

The Kp index (https://kp.gfz-potsdam.de/en/), measures the solar particle radiation by its magnetics effects on the Earth, and is estimated using data from observatories located at geomagnetic latitudes between 44° and 60° (either north or south). Global geomagnetic activity is measured in 3-hourly intervals and rated on a scale from 0 to 9. The Kp index during the May 2024 storm reached its maximum possible value of 9.0 between 00:00 and 03:00 UTC and between 09:00 and 12:00 UTC on 12 May (Fig. 1c).

## Comparing magnetometers and broadband seismometers

As shown in previous contributions^8^ signals related to geomagnetic storms can be detected seismically for frequencies beneath 0.01 Hz, in the range of the ultra-low frequency (ULF) magnetic pulsations, the ground manifestation of ultra-low frequency hydromagnetic waves propagating in the magnetosphere. ULF pulsations are classically classified according to their frequency^9^. Pc3 pulsations correspond to frequencies around 20–80 mHz, are observed regularly at middle latitudes and have been linked to daytime auroral pulsations^10^. Pc4 pulsations (7–22 mHz) are abundant, peak at latitudes below 60º and have been linked to ion-beam instability in the solar win^11^. Pc5 pulsations, ranging from 1.5 to 5 mHz, are the the easiest ULF waves to observe and have high amplitudes during magnetic storms^12,13^.

Pc3 pulsations are difficult to observe in seismic data as the signal in this band is strongly affected by surface waves generated by moderate/large magnitude earthquakes.  In the case of the May 2024 storm, three moderate magnitude earthquakes have been recorded worldwide before, during and after the geomagnetic storm (Taiwan 10/5 07:45, Mw 5.8; Mexico 12/5 11:39, Mw 6.4; Kermadec Islands 12/5 18:43, Mw 5.9). Furthermore, vibrations generated by oceanic waves interactions generate large seismic amplitudes in the 0.04–1.0 Hz band, with amplitude peaks at 0.07 and 0.14 Hz^14,15^. Seismic signals in the Pc4 band may include the low frequency signals generated by large seismic events and the continuous oscillation generated by oceanic infragravity waves, often referred as Earth’s hum^16,17^. Below 2 mHz, in the frequency band corresponding to the Pc5 pulsations, seismic amplitude variations have been related to atmospheric gravitational effects^18^. In our case, these effects do not seem to be relevant, since the seismic data filtered between 1.5 and 5 mHz have a good correlation with the magnetic data. Therefore, we decided to filter the seismic data in this frequency band, relating the amplitude variations observed in the seismic data to the Pc5 pulsations generated by the magnetic storm.

**Fig. 2:**
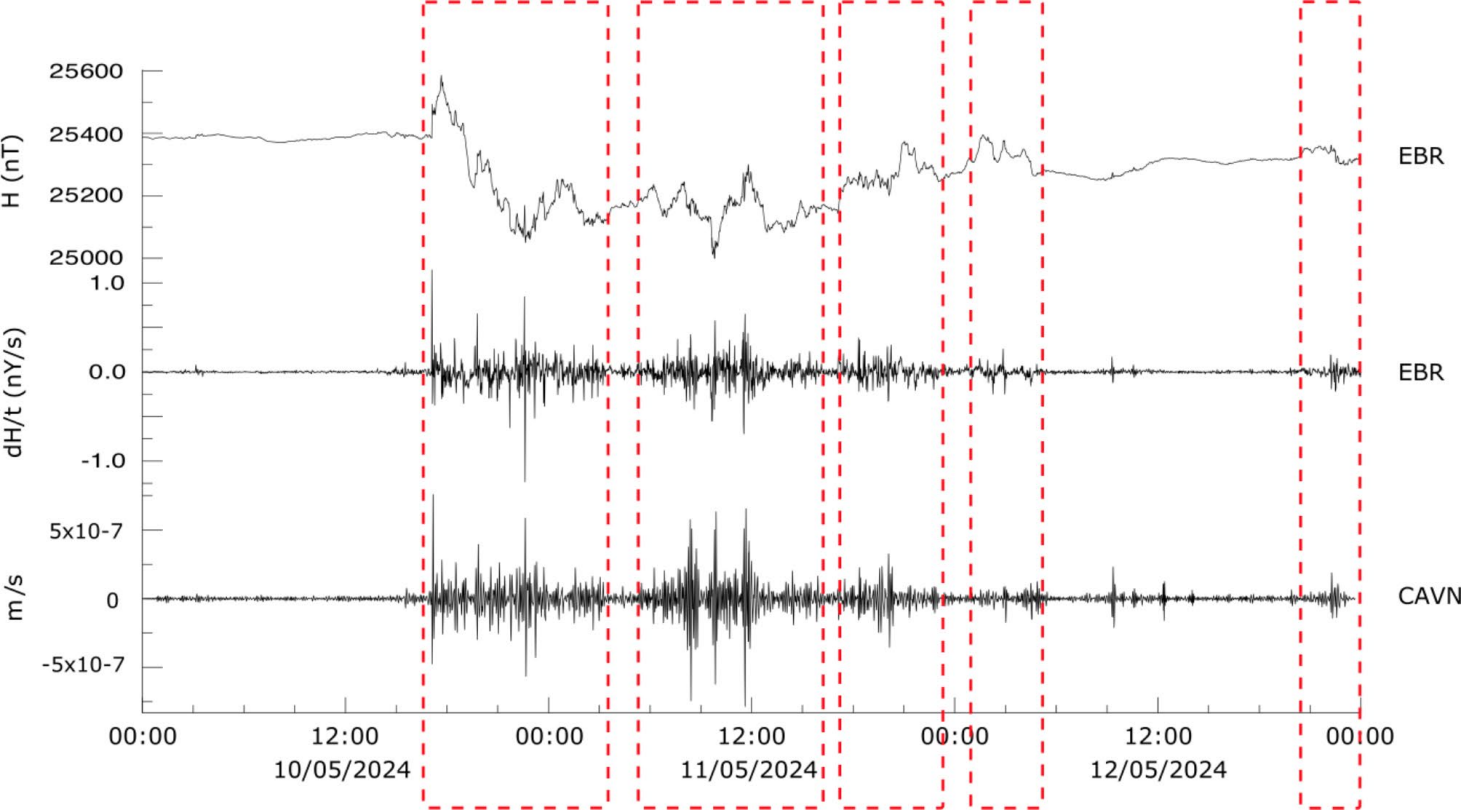
Horizontal magnetic intensity recorded at the EBR magnetometer, NE Spain (H, upper trace), variation of H over time at the same station (dH/t, middle trace) and broadband seismic data filtered between 1.5 and 5 mHz recorded at the CAVN, located 120 km from the EBR (lower trace). The red boxes highlight the different phases of the storm that are analyzed in the text.

First, we have investigated the available seismic data at a regional scale, focusing on the Iberian Peninsula, a mid-latitude area covered by different broadband seismic networks providing good spatial coverage. We have recovered the vertical component of the seismic data acquired by the Spanish national network (network code ES), the Portuguese national network (code PM) and the Catalan network (code CA), providing a denser coverage of NE Iberia. The raw seismic data have been corrected for the instrumental response following standard procedures and expressed as ground velocity. This procedure removes the effect of the recording instrument, allowing the effective ground motion to be measured. The signals are then band-filtered by applying a Butterworth filter with corner frequencies at 1.5 and 5 mHz. The decision on the presence or absence signals generated by the geomagnetic storm in the broadband seismic data is based on their consistency with the magnetograms available in the area. Sobolev et al.^19^ have shown that the seismic data are easier to correlate with the ratio of change of H, dH/t, than with the horizontal magnetic intensity. This is confirmed by the comparison between H and dH/t measured at the EBR magnetic station and the filtered seismic data recorded at the CAVN station, located 120 km from EBR (Fig. 2). The overall correlation between dH/t and the seismic data is clear, proving that seismic data can be used to describe the temporal evolution of the geomagnetic storm. During the time intervals with large amplitude signals related to the geomagnetic storm (red boxes in Fig. 2), Pearson’s correlation coefficient between the EBR magnetometer and the CAVN seismometer is around 0.7. This value is remarkable if we consider the different type of signals (magnetic field vs. seismic), the low sampling rate (1 sample per minute) and the distance between the two instruments (120 km).

Seismic and dH/t data show two main phases of the storm. The first phase starts at 17:05 with the SC and ends at 03:20 on 11 May, 10:25 h later. After a two-hour break, the second phase starts at 05:30 and lasts until 15:45, although its amplitude decreases significantly from 12:30 onwards. A third phase can be identified between 17:20 and 23:10 on 11 May and a later, less energetic phase between 01:45 and 05:30 on 12 May. During 12 May the amplitude is small, although some energetic peaks can be identified around 09:00 and 12:30. Finally, a later period of activity can be identified between 22:00 and 23:25. During the first phase, extreme values are observed at 17:05 on 10 May, corresponding to the SC, and at 22:35 on 10 May, coinciding with the local minima of the horizontal magnetic intensity. Additional peaks are identified at 17:40, 18:40, 19:50, 20:35, 21:05 and 21:45. During the second phase of the storm, three peaks with amplitude similar to the SC can be observed at 08:25, 09:50 and 11:35 on 11 May.

**Fig. 3:**
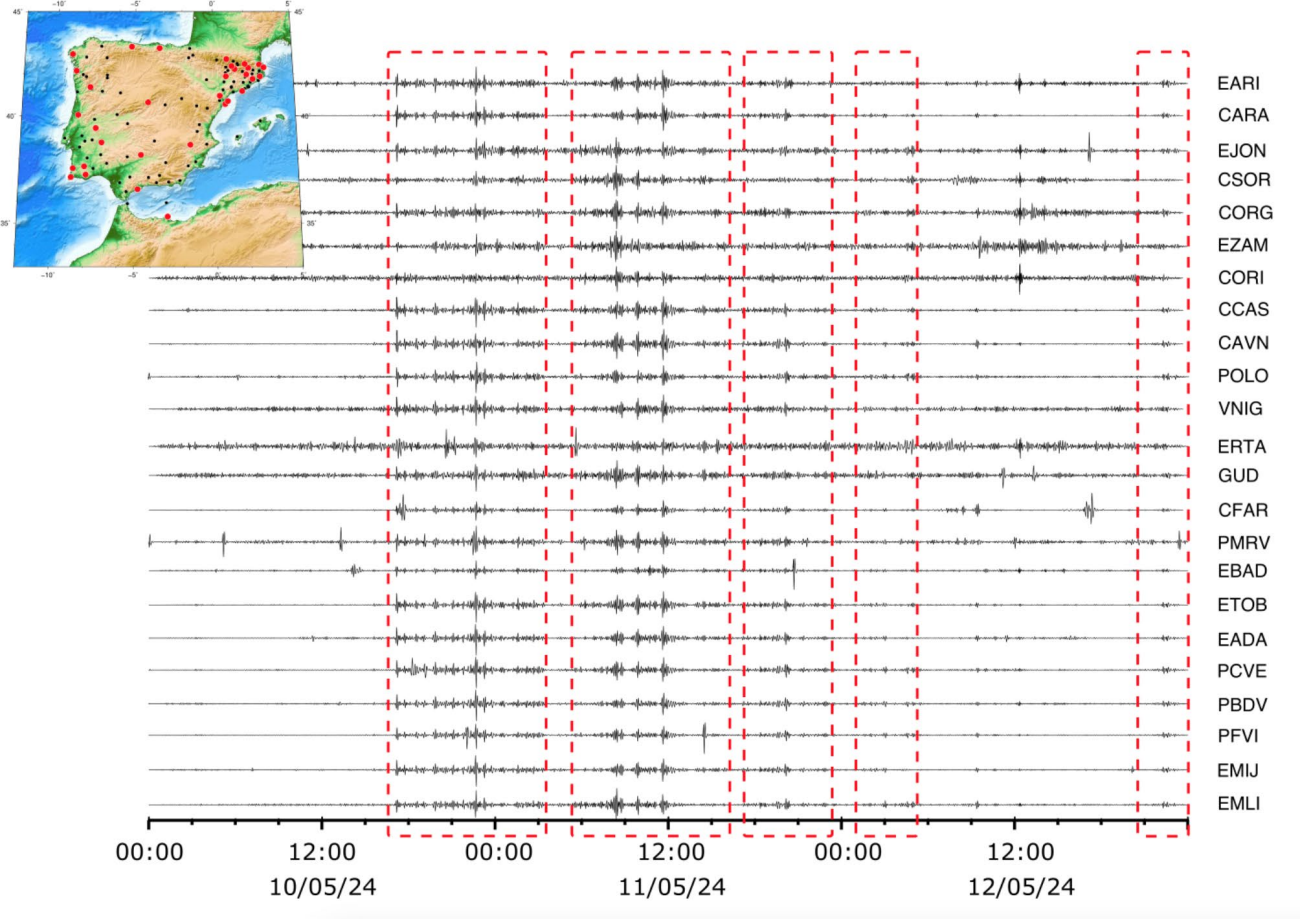
Seismic records, filtered between 1.5 and 5 mHz, during the May 2024 geomagnetic storm as recorded by broadband stations in the Iberian Peninsula. The red boxes show the different phases of the storm that are analyzed in the text. The amplitudes of each trace is represented using a different scale.

Figure 3 shows the seismic records of all the broadband seismometers deployed over Iberia with positive identifications of signals related to the May 2024 geomagnetic storm, representing around 20% of all the surveyed stations. This means that the detection of geomagnetic signals in seismic data is not a widespread phenomenon, but it cannot be considered an exceptional feature either. The seismic stations with positive identifications are distributed throughout the Iberian Peninsula and do not seem to be affected by geographical or geological variations. The identification of distinct storm phases discussed above is consistent with all the seismic data acquired in the Iberia Peninsula.

## Continental scale results

To gain a broader view of the existing seismic records of the May 2024 geomagnetic storm, we have collected the available seismic data over Europe, covering latitudes from 78ºN to 35ºN, between 00:00 on 10 May and 00:00 on 13 May. We have first used the LHZ channels, as these data, sampled at one sample per second (sps), are convenient for investigating low-frequency signals over periods of several days and can be efficiently processed. The data have been accessed using the different data servers integrated in the EIDA-EPOS (https://www.epos-eu.org/tcs/seismology/services/orfeus-observatories-and-research-facilities-european-seismology) facility and including data from Austria, France, Hungary, Italy, Denmark, Germany, Greece, Slovenia and Switzerland, as well as data from global and regional networks such as MedNet, GEOFON or Geoscope (network codes BW, CH, DK, FR, G, GE, GR, GU, HL, HU, IV, MN, OE, SL). As many seismic networks do not allow access to low-sampled data, we have completed our dataset by retrieving data recorded at the usual sampling rate of 100 sps. Working with this sampling requires additional resources to download and process the large volume of data involved. However, this has made it possible to complete the seismic picture of Europe, better covering areas such as Scandinavia or Iberia. We have in this way retrieved data from Germany, Great Britain, Greece, the Netherlands, Norway, Portugal, Spain and Sweden (grid codes CA, ES, GB, GR, HP, NL, NO, NS, PM, UP), using again the facilities at the different data centers integrated in the EIDA-EPOS facility. In the case of networks that include dense seismic arrays (such as the ARES and NORES arrays of the NO network, the G8 array of the NL network or the Gräfenberg array of the GR network) we have kept only one station of each array, to avoid over-representation of these areas in the final image.

Metadata provided by the seismic networks have been used to correct the instrumental response and represent the data in velocity (m/s), following the standard procedure used in seismological studies, restricting our study to the analysis of the vertical component of the seismic data. Out of a total of 845 stations surveyed, signals correlated with the geomagnetic storm have been identified in 311 cases, 37% of the total. In line with the results in the Iberian Peninsula, this detection rate in permanent seismic networks in Europe demonstrates that the detection of geomagnetic variations in broadband seismic data is not generic but no exceptional either.

**Fig. 4:**
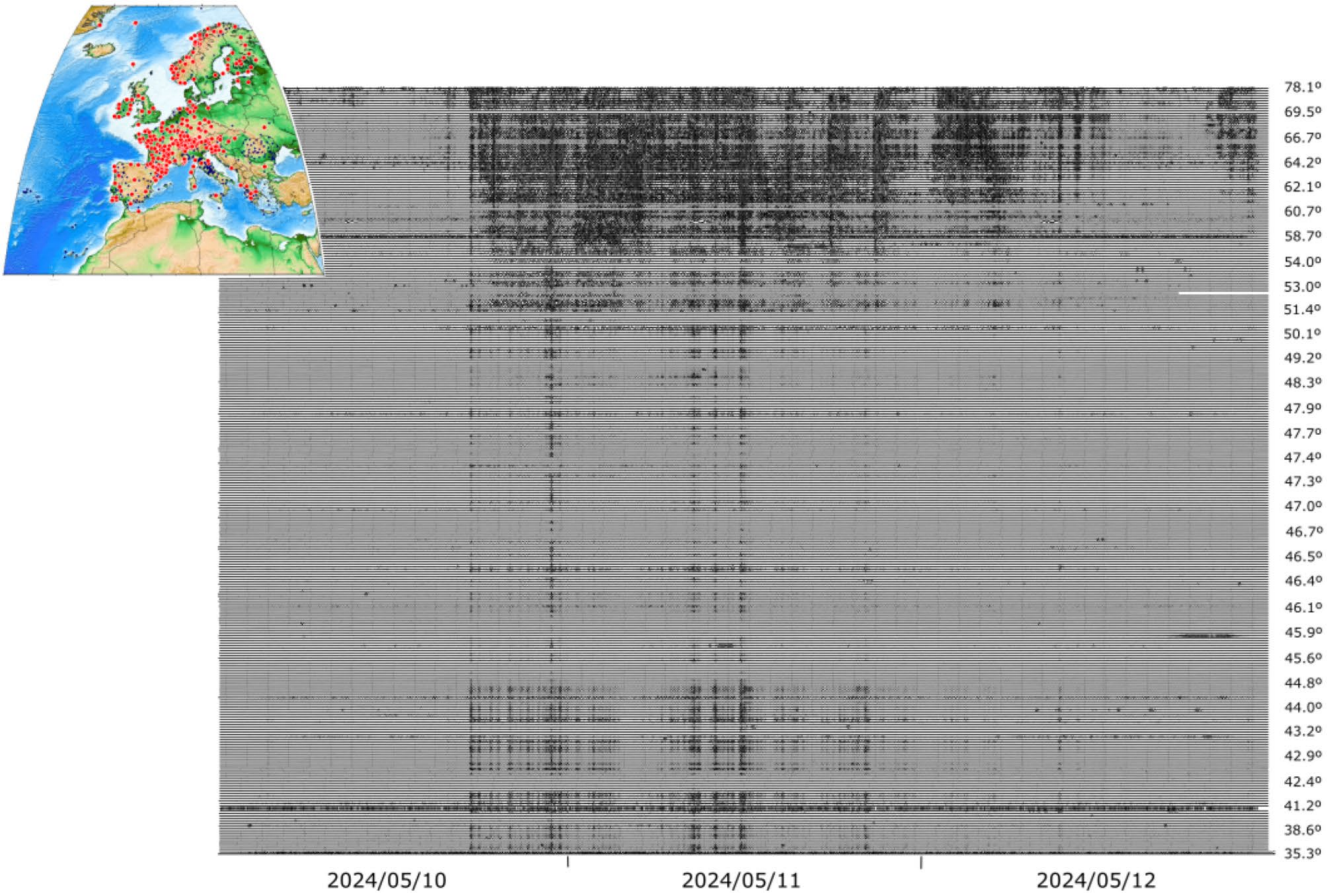
European broadband stations clearly showing the effects of the magnetic storm. The seismic data have been filtered between 1.5 and 5 mHz and plotted using a common amplitude scale. The labels on the right show the latitude of the corresponding trace. The inset map shows, with red dots, the sites with positive identifications and with black dots those where the geomagnetic storm did not affect the data.

Figure 4 shows the signals related to the May 2024 geomagnetic storm in Europe, filtered between 1.5 and 5 mHz, plotted using the same amplitude scale and sorted by the latitude of each site. As expected, stations located at higher latitudes show large amplitudes and more complex signals. Most stations below 50ºN show a similar pattern, with two main phases of amplitude increase followed by shorter duration episodes, in line with what was discussed for stations in the Iberian Peninsula. Interestingly, stations located at latitudes below 45ºN show a relative increase in energy compared to those in central Europe. As discussed below, the geographical distribution of stations with positive identifications is far from uniform. It is worth noting that, for the same region, there are 37 magnetic stations available at the INTERMAGNET facility, almost an order of magnitude less than for seismic data. Supplementary Fig. S2 shows the temporal variation of these magnetograms using the same procedure as in Fig. 4, allowing a comparison of the density of both types of data.

**Fig. 5:**
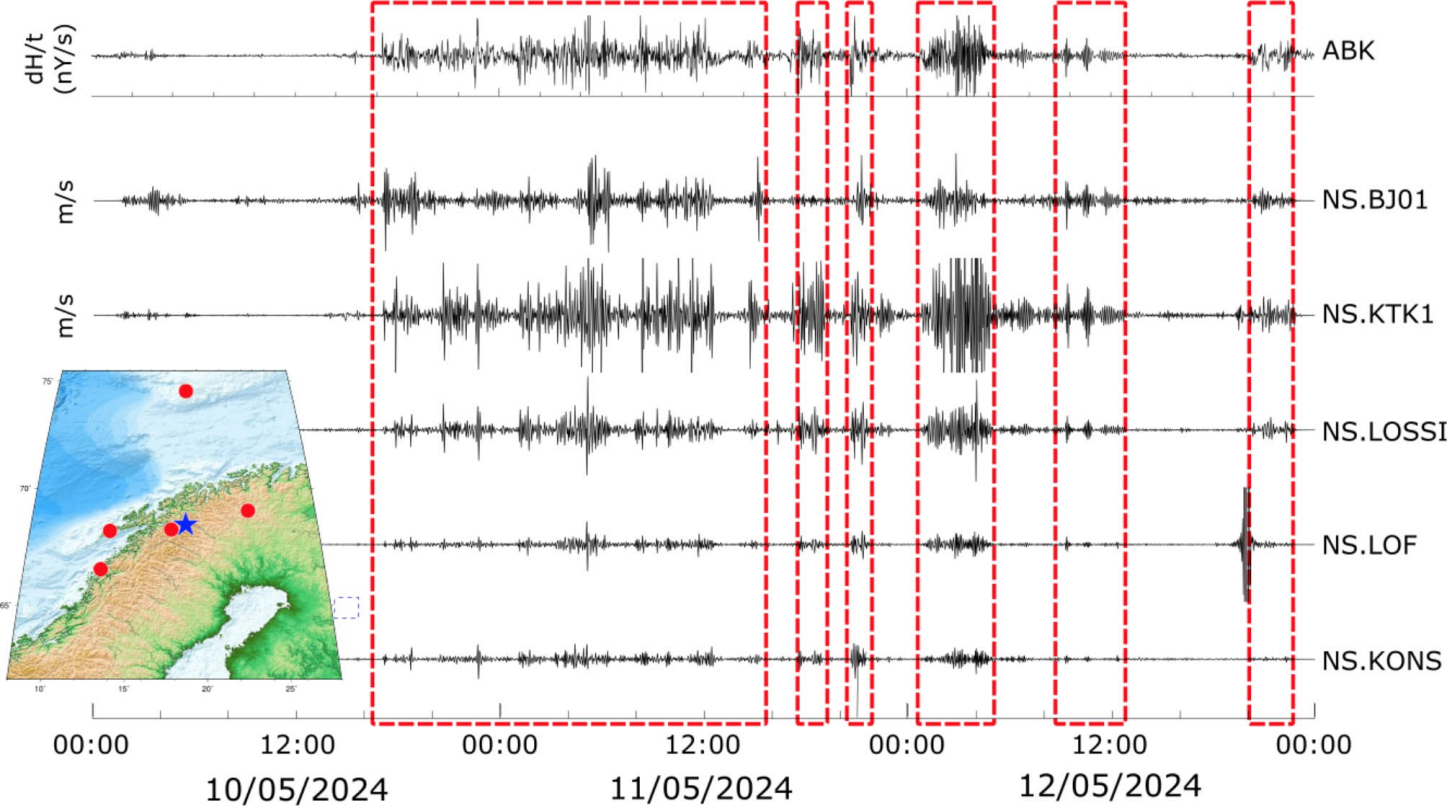
dH/t at the INTERMAGNET station Abisko (ABK, upper trace) and seismic records, filtered between 1.5 and 5 mHz for some of the stations of the NS array. The inset map shows, with red dots, the location of the seismic stations and with a blue star the location of the magnetic station. The red boxes highlight the different phases discussed in the text.

It is interesting to focus on the results from stations located at high latitudes, where the signals appear to be more complex. Figure 5 shows seismic data from five stations of the NS array in Norway, together with the magnetogram recorded at the ABK INTERMAGNET station (blue star in the map inset).

The first phase of the storm appears to extend here from 17:05 to 15:30 on 11 May, including the first two phases identified at mid-latitude stations. Later, large amplitudes appear between 17:30 and 19:00 and, with greater amplitude, at a peak around 21:00 that is not observed at mid-latitude stations. An interval with large amplitudes is identified between 01:00 and 04:00 on 12 May, which coincides in part with mid-latitude observations. Intervals of less pronounced activity can be identified between 07:00 and 13:00 and 20:00 and 23:00 on 12 May. This last phase has been identified at mid-latitude stations, while during the interval from 07:00 to 13:00, only isolated peaks are observed at mid-latitudes.

## Global scale results

To complete the review of the effects of the May 2024 storm on broadband seismic instruments, data have been retrieved from broadband seismic stations distributed around the world and integrated into major large-scale global seismic networks, including the IRIS/IDA and IRIS/USGS components of the Global Seismographic Network (network codes II and IU), Geoscope (network code G) and GEOFON (network code GE). For those sites with multiple sensors, we have retained those marked with location code 00, which is the primary sensor. As in the previous cases, the raw seismic data have been corrected for the instrumental response, expressed as ground velocity and bandpass filtered between 1.5 and 5 mHz.

**Fig. 6:**
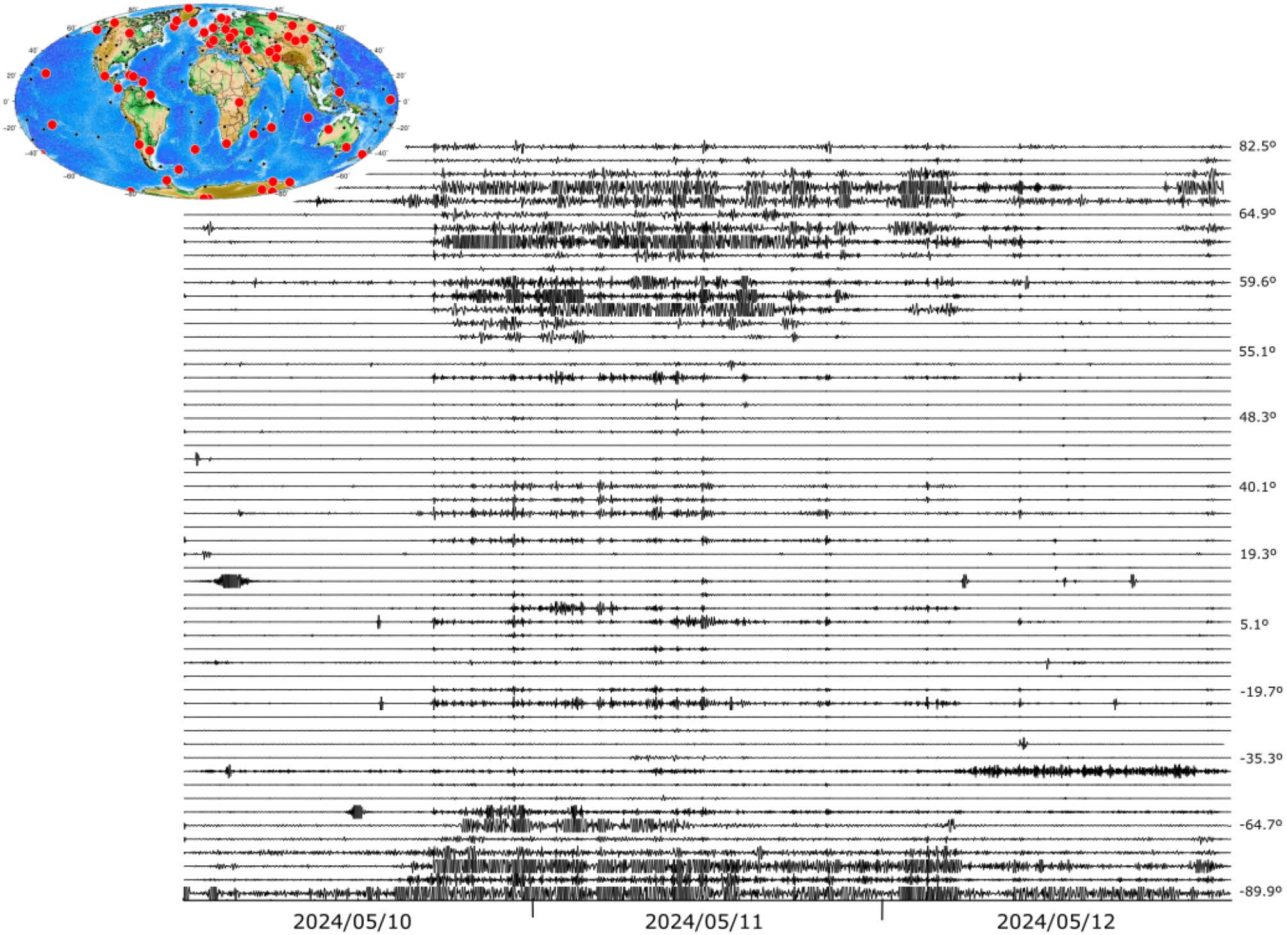
Seismic record of the May 2024 storm at broadband stations around the world, filtered between 1.5 and 5 MHz and represented using a common amplitude scale. Labels on the right show the latitude of the corresponding trace. Red dots on the inset map show the location of positively identified sites, while black dots show those located where the geomagnetic storm did not affect the data.

Clear signals related to the magnetic storm have been identified at 57 of the sites, representing 39% of the total, in the same range as for the European scale section (Fig. 6). The detection rate is similar for stations in both hemispheres, as well as for stations on islands in the middle of large ocean basins. It is noteworthy that only a few stations in North America appear to be sensitive to the geomagnetic storm.

**Fig. 7:**
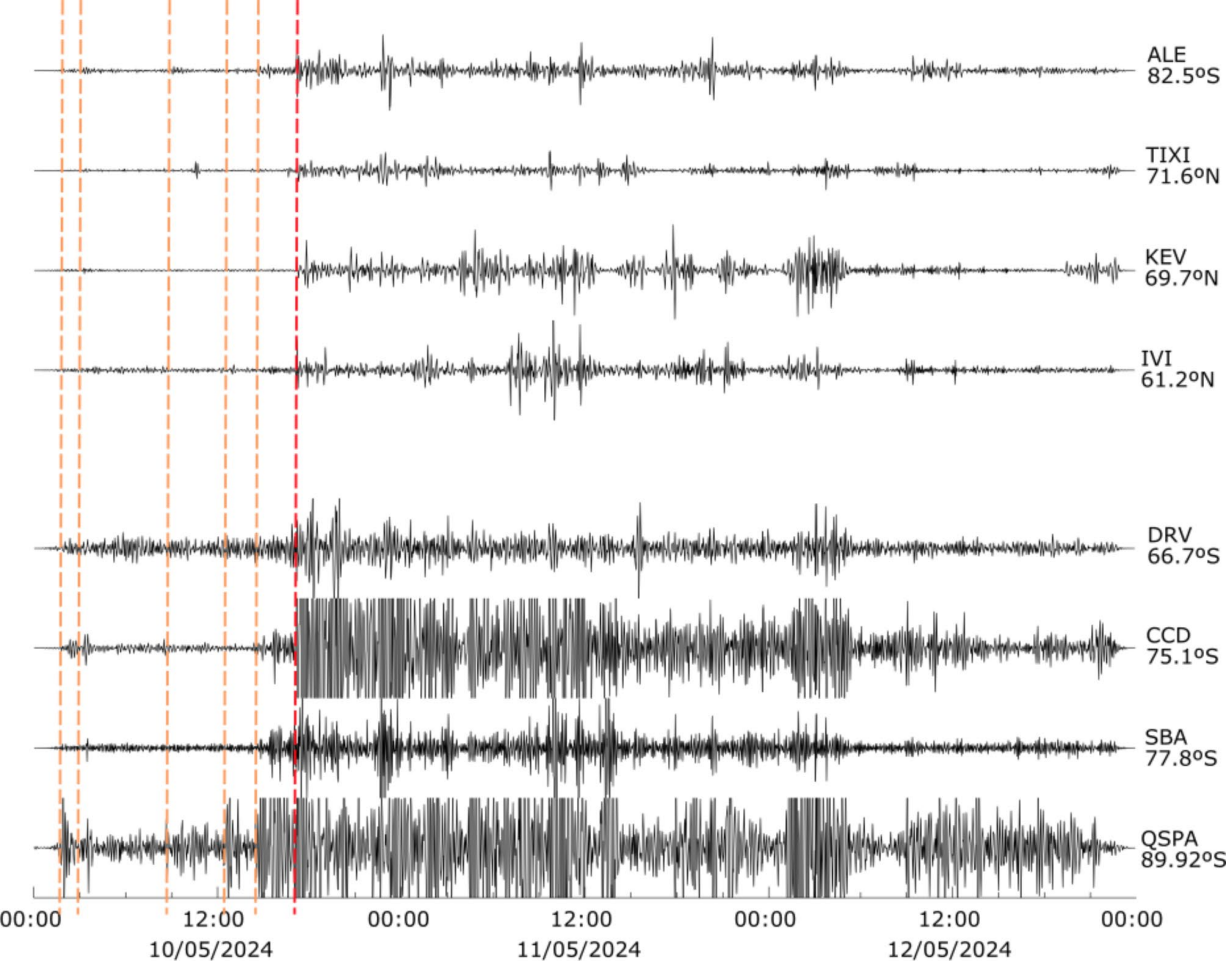
Seismic record of the May 2024 geomagnetic storm at broadband stations in the Arctic and Antarctic, filtered between 1.5 and 5 mHz and represented using a common amplitude scale. The orange dashed lines show the identified signals preceding the seismic storm (red dashed line).

The mid-latitude stations in the southern hemisphere show a very similar pattern to that discussed for Europe. Larger than usual amplitudes are observed at stations located beyond 60º in both hemispheres, although differences can be noted between the Arctic and Antarctic areas (Fig. 7). In the Arctic, the northernmost stations with positive identifications, located in the range 70º-82ºN, show amplitudes that are clearly lower than those stations located in the range 60–70ºN. In contrast, all seismic stations below 60°S, including the station at the South Pole, show large amplitudes as well as clear signals preceding the arrival of the SC by several hours, including peaks at 01:50, 03:10, 09:00, 12:30, 14:40 and 15:40 on 10 May 2024.

As discussed in the next section, data from the magnetometer installed at the QSPA site at the South Pole confirm that such signals are related to geomagnetic storm activity. Although with a lower amplitude, most of these peaks can also be identified at seismic stations in the Arctic region. Therefore, seismic stations at high/low latitudes can detect magnetic disturbances preceding the SC.

## Discussion on the origin of the signals

Previous contributions have pointed out that records from broadband seismic stations can be affected by different types of electromagnetic signals, including magnetic storms, geomagnetic pulsations, auroral electrojets, lightning, magnetic fields produced by local supply currents, disturbances due to the passage of moving magnetic or electric elements or leakage currents associated with transportation systems^5–8,20^. Early evidence of the interaction between the magnetic field and broadband seismic sensors was obtained in Stuggart by Professor Erhard Wielandt, who noticed a decrease in the amplitude of long-period signals recorded by the STS-1 broadband seismometer prototype during a strike by electric tram workers (reported in^6^).

Recently, Sobolev et al.^19^ have proposed that the signals recorded by broadband seismometers during magnetic storms may reflect the conversion of the electromagnetic field to elastic energy through the piezoelectric effect in the lithosphere, a point that will explain the lack of observations on deep-sea islands. However, most authors relate the presence of magnetic signals in broadband data to the physical interaction between the Earth’s magnetic field and parts of the broadband seismic sensor, resulting in apparent motions which are not related to Earth’s shaking. The raw measure provided by modern broadband sensors is the current intensity applied by the force-balance system to keep a reference mass stationary. This current intensity can be modified if variations in the Earth’s magnetic field are capable of inducing currents in the feedback coil of the electromagnetic force transducer of the seismometer^5,20^. The magnetic signals in broadband seismometers may also be due to the magnetization of the suspension springs used in the sensors^8^, which are built using Elinvar, an alloy that has the required small thermal coefficient but is sensitive to magnetic fields.

Magnetic field variations due to magnetic storms produce the so-called Geomagnetic Induced Currents (GICs) in conductors operating at or near the Earth’s surface^21^. Although the largest magnetic field variations associated with those events are observed at high latitudes, GICs are also recorded at mid-latitudes during major storms^22^. It is well known that GICs may be strong enough to degrade voltage power transformers, increase corrosion of pipelines steel or disturb seabed fiber optics systems^23^. It seems plausible to propose that GICs may also be the responsible of the magnetic signals recorded in some broadband stations. In this regard, Diaz et al.^5^ have shown that currents induced by the metro transportation system in Barcelona generate signals at frequencies below 0.01 Hz at a broadband seismometer located within the city.

We have seen that seismic data at a relevant percentage of sites can be correlated with the temporal variation of the horizontal magnetic field. However, we have not so far discussed questions such as why the geomagnetic storm affects only some broadband stations or whether the signals in the seismic records are consistent at a local scale. To address these questions, we have inspected the available data in two particular cases. On the one hand, we have explored the data available at the QSPA site, located at the South Pole and equipped with a magnetometer and different models of broadband stations installed at different depths. On the other hand, we have inspected the data from a classical seismic , the Gräfenberg array in southern Germany, made up of several broadband stations separated by a few kilometres and covering an area of about 100 × 40 km. Finally, we will discuss the variations in the degree of detection of geomagnetic storm signals between different large-scale seismic networks.

### Multiple broadband seismometers at the South Pole QSPA station

**Fig. 8:**
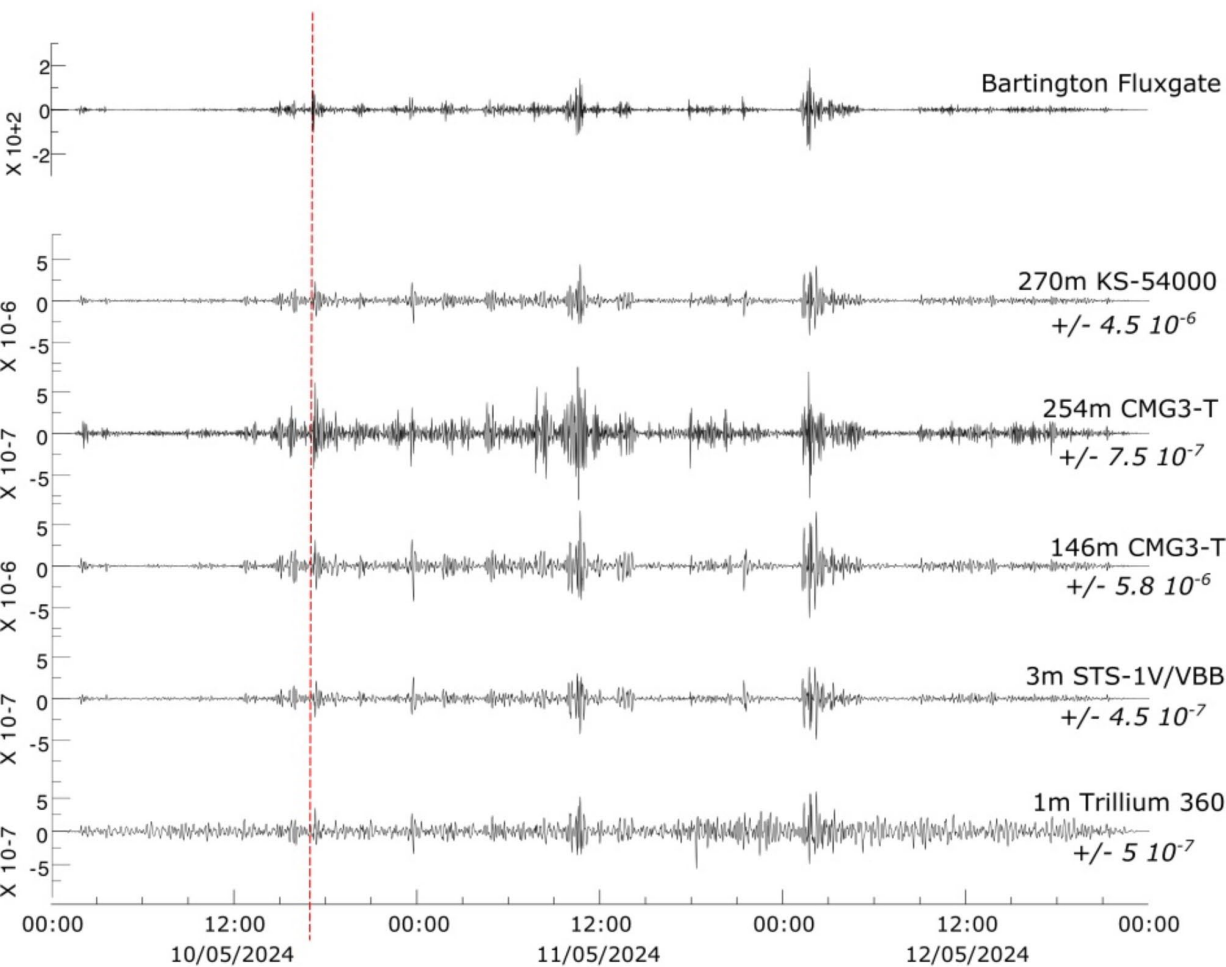
Upper trace: Time variation of the horizontal magnetic field (dH/t) at the Bartington magnetometer installed at the QSPA site. Lower traces: Seismic data filtered between 1.5 and 5 mHz recorded at the different broadband sensors installed at the QSPA site. The labels on the right show the depth and type of each of the sensors (top) and the amplitude range of the trace (bottom). Note that each trace is represented using a different amplitude scale, which is shown on the labels on the left side.

The QSPA station is located at the South Pole Earth Science Remote Observatory, 8 km from the geographic South Pole (89.929ºS, 144.438ºE). The station, integrated into the IU seismic network, is considered one of the quietest seismic stations in the world. Its equipment includes, among other sensors, three broadband instruments installed in boreholes at depths of 270 m (Kinemetrics KS54000), 255 m (Güralp CMG3-T) and 146 m (Güralp CMG3-T), a Streckeisen STS-1 V/VBB sensor installed in a cylindrical dome on the ground, 3 m below the snow surface, and a Nanometrics Trillium 360 sensor installed 1 m below the surface. A Bartington fluxgate is also available to measure local variations of the magnetic field (Fig. 8).

The first observation is that the signals of all broadband sensors installed at the QSPA site show signals in the 1.5–5.0 mHz band which are clearly consistent with the temporal variation of the horizontal magnetic field recorded by a magnetometer placed at the same location. However, significant differences can be detected between the different instruments. The two deepest sensors, located at similar depths (270 and 254 m), differ in amplitude by a factor of 6 , with amplitudes in the range +/- 4.5 10^− 6^ m/s on the deepest one, a KS-54000 sensor, and amplitudes between +/- 7.5 10^-7^  m/s on the CMG3-T sensor. The two CMG3-T sensors located at different depths show large amplitude differences, +/- 7.5 10^-7^ m/s on the sensor buried at 254 m versus +/-5.8 10^− 6^ m/s on the sensor at 146 m depth. This fact is remarkable, since electromagnetic signals are expected to attenuate with depth following an exponential law. The STS-1 V/VBB sensor has the lowest amplitudes, similar to those on the CMG3-T installed at 254 m, and very close to those recorded by the Trillium 360 sensor, despite being installed close to the surface. To verify that these differences do not arise from errors in the response files, we have extracted the body wave arrivals for the magnitude 6.4 earthquake with epicenter in Mexico that occurred on May 12, 2024 at 11:39 (Supplementary Fig. S3). No differences in amplitude or polarity can be observed between the different broadband sensors, contrary to what was observed in the recordings acquired during the magnetic storm.

These large amplitude differences between instruments located in one of the quietest places in the world confirm that the sensitivity of broadband instruments to magnetic events is related to the technical characteristics of each instrument and, in particular, to the magnetic isolation system. The technical specifications of the Nanometrics Trillium360 sensor list its low magnetic sensitivity (0.01 m/s2/T) as one of the instrument’s strong points^24^; data acquired during May 2024 at QSPA confirm this point and highlight the role of magnetic isolation systems.

### Gräfenberg array data analysis

The Gräfenberg array in southern Germany was installed in the mid-1970s and has been one of the first examples of a broadband seismic array designed for the observation of global events. The array extends about 100 km in a north-south direction and 40 km in an east-west direction. It is formed by13 stations equipped with Streckeisen STS2 broadband seismometers and spaced 10–15 km apart. We decided to explore the data acquired by this classic array, with nearby stations equipped with the same equipment, to check whether the May 2024 storm was recorded consistently across the array.

**Fig. 9:**
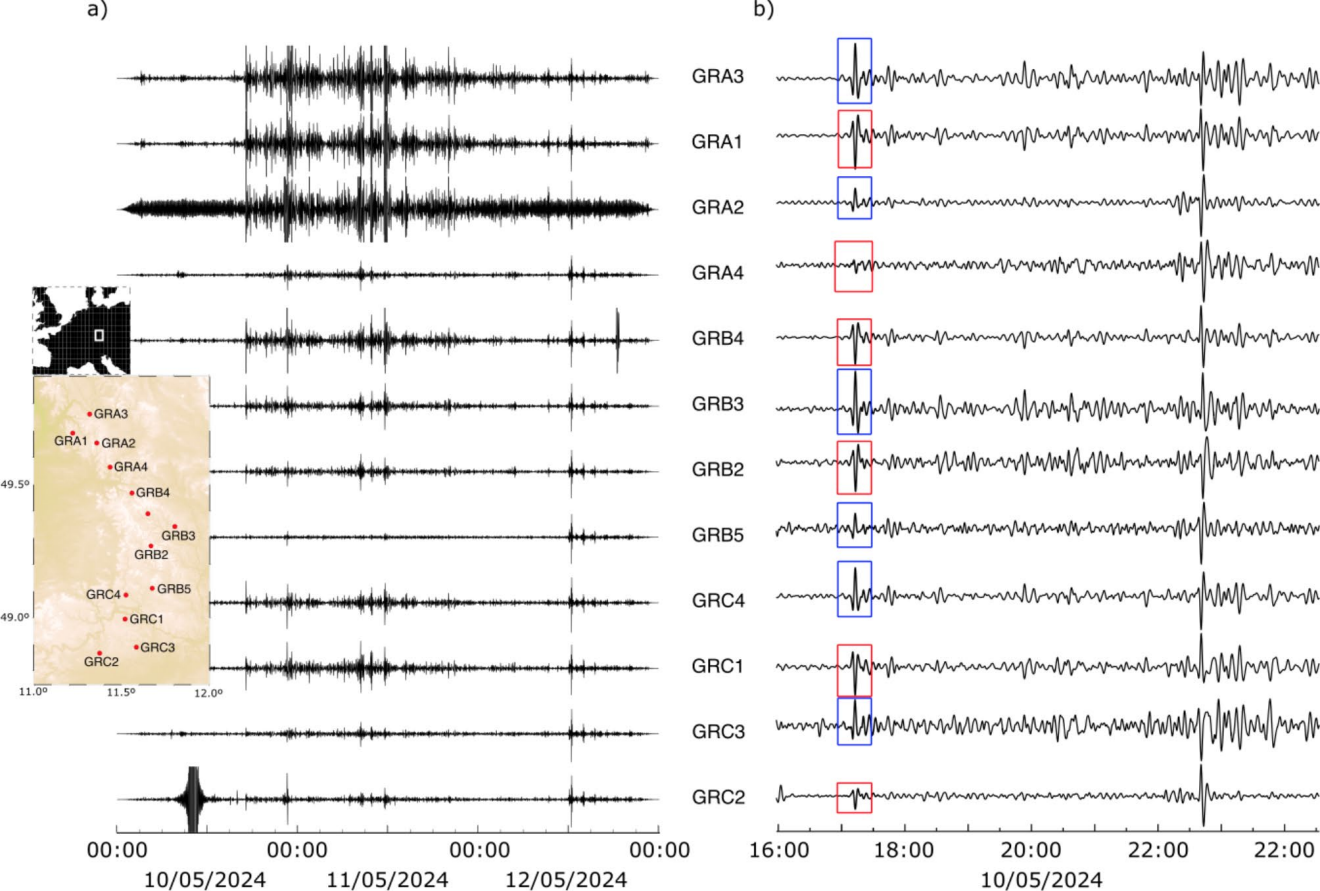
Seismic data from stations in the Gräfenberg array, filtered between 1.5 and 5 mHz. (**a**) Traces from the three days of maximum geomagnetic storm activity, plotted using the same amplitude scale for all traces. (**b**) Zoom in on the first few hours of the storm, now plotted using a normalized amplitude scale. Blue and red squares highlight polarity differences in the SC signal records.

Figure 9a shows the seismic data filtered between 1.5 and 5 mHz during the three days of maximum solar activity, with all traces represented on the same amplitude scale. As can be seen, all available stations recorded the magnetic signals, but significant differences in amplitude can be noted. The three northernmost stations have amplitudes greater than the rest by a factor of 5. The difference between GRA2 and GRA4, separated by only 11.7 km, is particularly noteworthy. Significant amplitude variations can also be observed at the stations in the south of the array. Figure 9b shows the same data zoomed in on the first hours of the geomagnetic storm and represented using a normalized amplitude scale, in order to verify eventual polarity changes in the data. As can be seen, the polarity of the signals changes between neighboring stations, such as between GRA3/GRA1, GRB4/GRB3 or GRC1/GRC3. As in the previous case, we have extracted the arrivals from the Mw 6.4 Mexico earthquake to verify that these differences are not related to problems in the response of the instruments (Supplementary Fig. S4).

Data acquired at the Gräfenberg array, with significant changes in both amplitude and polarity between broadband stations equipped with the same instruments and separated by distances of 10–15 km, suggest that these differences may arise from local variations in the electromagnetic field. In the last decade, wind turbines have been installed in the area around the Gräfenberg array, resulting in degradation of the array performance in different frequency bands^25^. We suggest that power lines installed to connect the wind turbines to the grid may generate local electromagnetic fields that interact with magnetic field variations due to the solar storm, explaining the observed amplitude and polarity variations.

### Magnetic sensitivity differences among seismic large scale networks

Inspection of seismic data at different scales has shown that there are striking differences in the sensitivity of different networks to magnetic field variations associated with the geomagnetic storm.

The data available in the Iberian Peninsula, presented in Sect. 2, include contributions from three different seismic networks, equipped with a variety of broadband sensors. For the PM network, covering Portugal, 15% of the surveyed sites (6 out of 39) show signals clearly related to the geomagnetic storm, most of them equipped with Nanometrics Trillium T120 sensors. None of the instruments equipped with mid-range sensors (LE-3D5s, LE-3D20s) detected magnetic signals. The CA network, covering NE Iberia, has a high detection rate of magnetic signals, which have been identified in 40% of the sites (8 out of 20). Most of the stations with positive detections are equipped with Trillium T120 (5 out of 8), while most of the sites without detections are equipped with CMG3T or STS2.5 (9 out of 12) sensors. The ES network has a detection rate of 17% (10 out of 58) and among the positive detections there are 5 Guralp CMG3T and 4 Nanometrics Trillium T120. Therefore, it is difficult to establish a direct relationship between the type of broadband sensor and its sensitivity to magnetic signals for this network, suggesting that other factors influence it, such as the installation method or the local electromagnetic field.

At the European scale, the most striking difference observed concerns the low sensitivity to the magnetic signals of the stations covering Italy (3% of positive detections, 2 out of 68), in particular compared to the high number of detections in France (54%, 92 out of 171). According to the published metadata, most of the stations in the IV network providing low sampling data (LHZ channels) are equipped with Trillium40s seismometers, which have a corner frequency of 25 mHz (40 s). Therefore, the lack of magnetic signals in the Italian seismic data can be explained by the frequency range of the instrumentation, which does not make it possible to record the low frequency ULF magnetic pulsations. Note that the only two of the IV sites with positive identifications are equipped with wider broadband instruments.

On a global scale, it is remarkable the low level of detections observed for the stations covering North America and, more particularly, the continuous continental part of the USA. In this area, we have recovered LHZ channels from 12 different sites, all of them equipped with STS-6A seismometers, with no signals related to the geomagnetic storm being observed at any of them. According to the technical information provided, the STS-6A seismometers include magnetic shielding for high latitude operations (https://kinemetrics.com/wp-content/uploads/2017/04/datasheet-360s-borehole-seismometer-sts6a-kinemetrics-streckeisen.pdf). Therefore, it seems that the lack of magnetic signals recorded on broadband seismometers in this area is due to the good magnetic shielding of the instrumentation.

All these observations show that the response of broadband seismometers to magnetic field perturbations depends on different factors, including the corner frequency of the instrument, the quality of the magnetic shielding, and the properties of the local electromagnetic field. The fact that signals related to magnetic field variations have been observed in broadband instruments manufactured by different companies, probably using different components and materials, does not argue in favor of an origin related to the magnetization of the suspension springs used in the sensors^8^. On the contrary, we believe that the observations of the May 2024 geomagnetic storm are consistent with the hypothesis that the currents induced by the magnetic perturbation add to the current intensity applied by the force-balance transducer of the seismometer to keep the reference mass fixed^20^. Since our results have shown that local variations in the electromagnetic field, due for example to the presence of nearby power lines, can affect the data, discrimination between both effects will probably require a laboratory study with controlled parameters.

## Conclusions

The May 2024 geomagnetic storm has been widely recorded on broadband seismic sensors distributed around the world. Magnetic signals associated with the solar storm can be clearly identified in seismic data over a time interval of more than 55 h, thus becoming one of the longest geomagnetic storms recorded by seismic instruments so far. The detection of geomagnetic signals in seismic data is not a widespread phenomenon, but cannot be considered an exceptional feature, as the rate of occurrence ranges between 35 and 50% for the seismic networks equipped with very broadband instruments. Magnetic signals recorded on seismic sensors can be detected for frequencies below 10 mHz, although they are better isolated in the 1.5-5 mHz frequency band, corresponding to the Pc5 pulsations. As the number of broadband stations is greater than that of magnetometers, seismic data retrieval allowsto refine the temporal and spatial evolution of these pulsations. As an example, in the European region more than 300 seismic traces recording the geomagnetic storm are available, compared to over 30 magnetograms. Consistent with the magnetic data, geomagnetic storm-related signals in seismic data have higher amplitudes at high latitudes. Inspection of the available data in the Arctic and Antarctic regions shows some signals that clearly precede the SC signature.

There are multiple factors determining whether a broadband seismic station is sensitive to magnetic field variations resulting from geomagnetic storms. As the ULF magnetic pulsations have frequencies mostly ranging between 1.5 and 20 mHz, they can only be detected by broad band seismic instruments with corner frequencies below 20 mHz (50s). Inspection of sites equipped with multiple broadband sensors and of seismic arrays with identical instruments located tens of kilometers apart has shown that the amplitude and polarity of the signals change significantly due to the properties of each instrument, but also due to the local electromagnetic field. No signals related to the May 2024 geomagnetic storm have been observed on most of the broadband sensors specifically designed to have good magnetic shielding, such as the Nanometrics Trillium 360 on Streckteisen STS6A. The recording of magnetic signals in broadband seismometers appears to be related to the interaction between the currents induced by variations in the magnetic field and the force balance transduced by the seismometer.

Using seismic traces to quantitatively model magnetic pulsation waveforms will require specific calibration of each instrument and a study of the electromagnetic field close to the station location. However, we have shown that seismic data can be used to describe the different phases of magnetic field variations induced by the geomagnetic storm of May 2024, comparing their relative amplitudes and their differences as a function of latitude. We are therefore convinced that broadband seismometers can significantly densify the data provided by the magnetic network, becoming an excellent tool to advance the understanding of the magnetic field disturbances associated to solar storms.

## Methods

To inspect the seismic data at global scale, we have selected a group of broadband seismic stations integrated in the main worldwide-scale seismic networks, including the IRIS/IDA and IRIS/USGS components of the Global Seismographic Network, as well as the Geoscope and Geofon networks. We have downloaded and processed the available data, restricting ourselves to the LHZ channels (vertical component with a sampling rate of 1 sample per second). For regional and local scales we have downloaded and processed low sampling data if available.

In all cases, the instrument response has been removed from the data using the standard procedures included in the Obspy package^26^. Standard band-pass Butterworth filtering has been applied using the SAC^27^ package, also used to plot the seismic traces.

## Electronic supplementary material

Below is the link to the electronic supplementary material.

Supplementary Material 1

## Data Availability

The magnetic data used is publicly available from the Intermagnet data portal (https://intermagnet.org/new_data_download.html). All the seismic data used in this contribution are publicly available using the EIDA-EPOS (http://www.orfeus-eu.org/data/eida/) and FDSN (https://www.fdsn.org/services/) data services. The code and DOIs of the seismic networks used are listed in Supplementary Material 1.
