# Author Correction: Monitoring May 2024 solar and geomagnetic storm using broadband seismometers

**DOI:** 10.1038/s41598-025-88222-x

**Published:** 2025-02-18

**Authors:** J. Díaz

**Affiliations:** GEO3BCN – CSIC, Barcelona, Spain

Correction to: *Scientific Reports* 10.1038/s41598-024-81079-6, published online 03 December 2024.

The original version of this Article contained errors in Figs. 4 and 6, where the dates in the x-axis were incorrectly stated as ‘2025/05/10’, ‘2025/05/11’ and ‘2025/05/12’, respectively. The correct dates are now stated as ‘2024/05/10’, ‘2024/05/11’ and ‘2024/05/12’, respectively.

The original Figs. 4 and 6 and accompanying legends appear below.

**Fig. 4:**
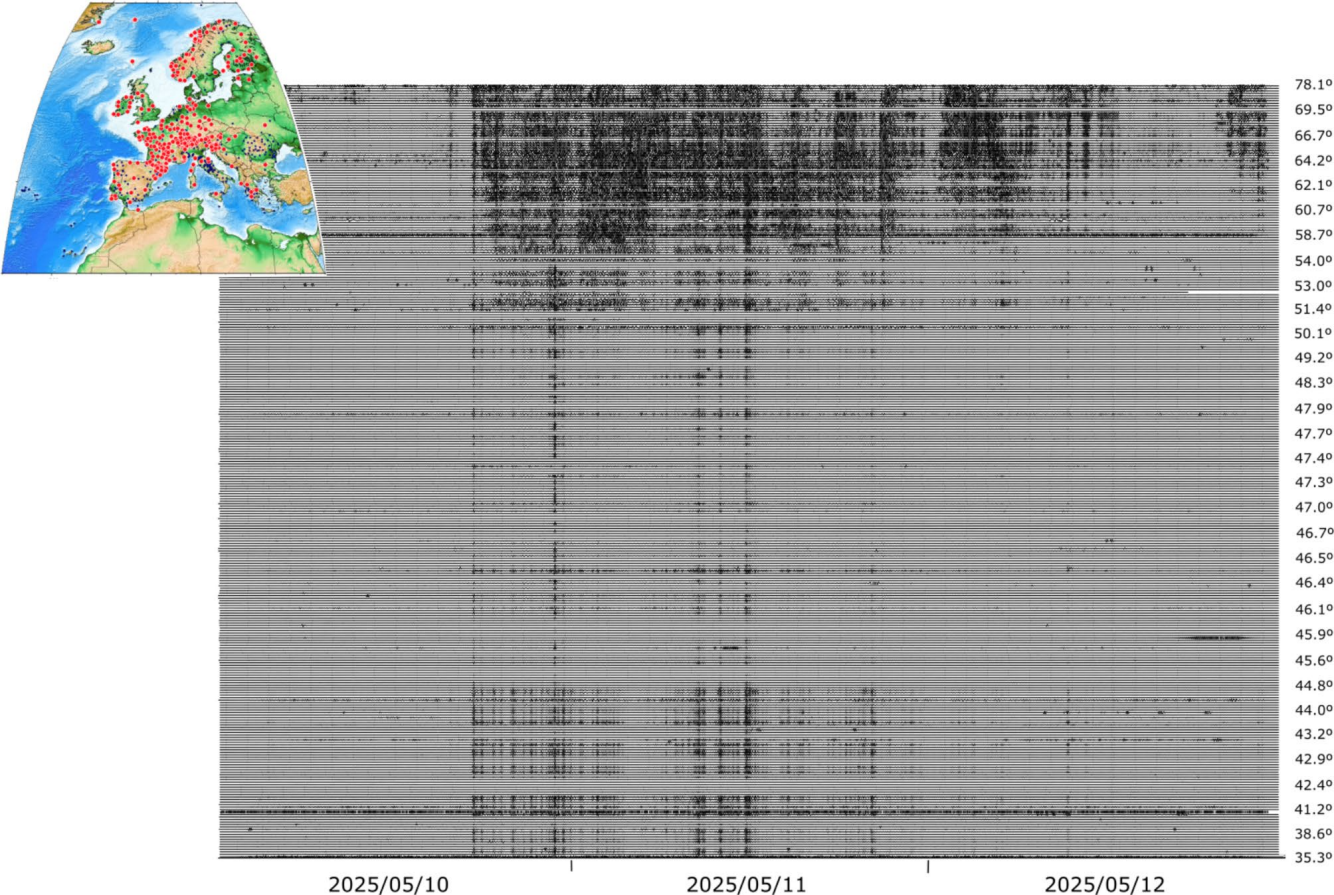
European broadband stations clearly showing the effects of the magnetic storm. The seismic data have been filtered between 1.5 and 5 mHz and plotted using a common amplitude scale. The labels on the right show the latitude of the corresponding trace. The inset map shows, with red dots, the sites with positive identifications and with black dots those where the geomagnetic storm did not affect the data.

**Fig. 6:**
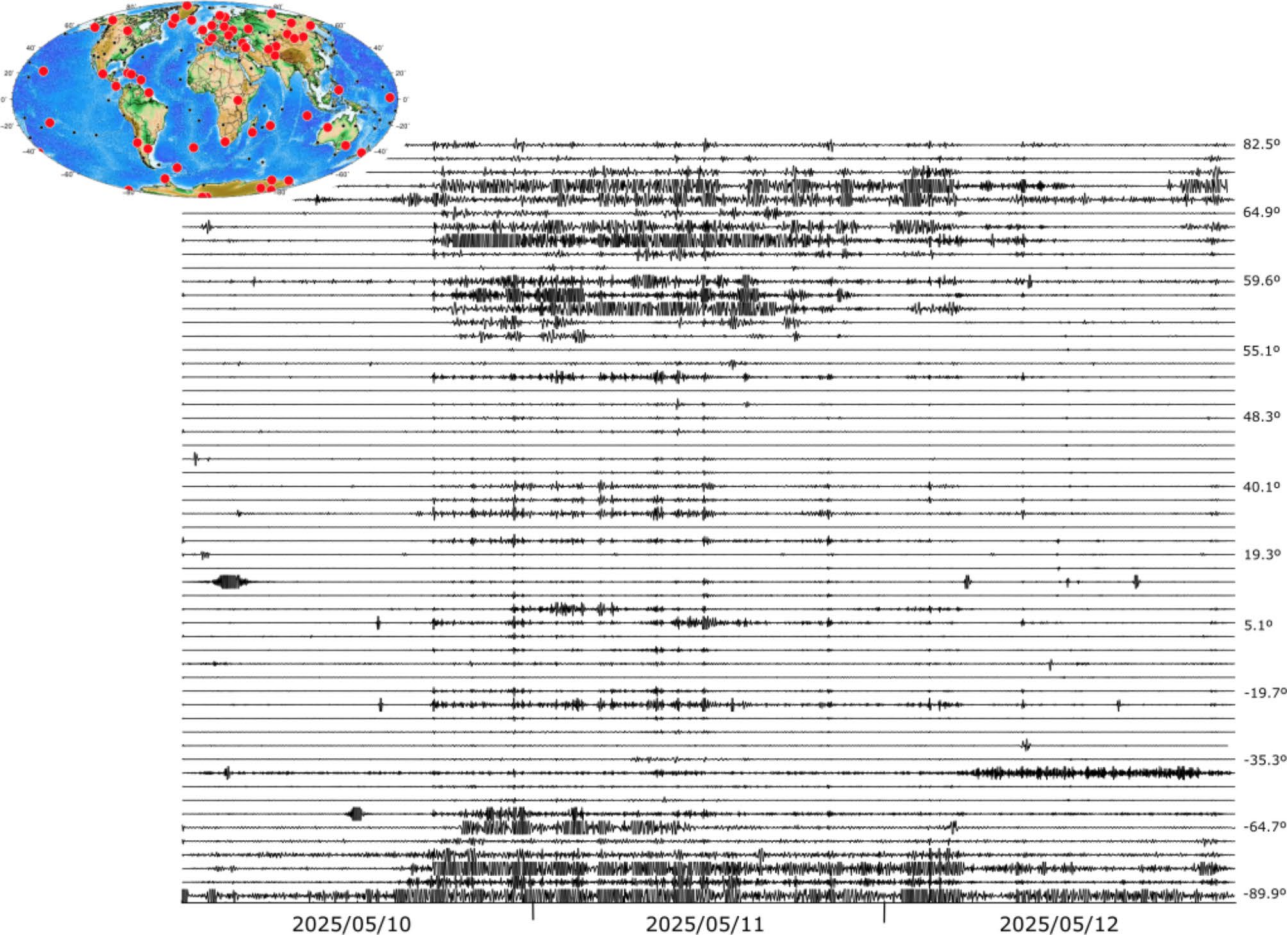
Seismic record of the May 2024 storm at broadband stations around the world, filtered between 1.5 and 5 MHz and represented using a common amplitude scale. Labels on the right show the latitude of the corresponding trace. Red dots on the inset map show the location of positively identified sites, while black dots show those located where the geomagnetic storm did not affect the data.

The original Article has been corrected.

