# Supplementary material for: Monitoring May 2024 solar and geomagnetic storm using broadband seismometers
Source: Sci Rep. 2024 Dec 3;14:30066. doi: 10.1038/s41598-024-81079-6 (PMC11615343; doi:10.1038/s41598-024-81079-6)

1. **Supplementary Material 1:** The codes, references and DOIs of the seismic networks used in this study are:

- BW, Department of Earth and Environmental Sciences, Geophysical Observatory, University of Munchen. (2001). *BayernNetz* [Data set]. International Federation of Digital Seismograph Networks. <https://doi.org/10.7914/SN/BW>
- CA, Institut Cartogràfic i Geològic de Catalunya. (1984). *Catalan Seismic Network* [Data set]. International Federation of Digital Seismograph Networks. <https://doi.org/10.7914/SN/CA>
- CH, Swiss Seismological Service (SED) At ETH Zurich. (1983). *National Seismic Networks of Switzerland*. ETH Zürich. <https://doi.org/10.12686/sed/networks/ch>
- DK, GEUS Geological Survey of Denmark and Greenland. (1976). *Danish Seismological Network* [Data set]. International Federation of Digital Seismograph Networks. <https://doi.org/10.7914/nw3x-df02>
- ES, Instituto Geográfico Nacional, Spain. (1999). *Spanish Digital Seismic Network* [Data set]. International Federation of Digital Seismograph Networks. <https://doi.org/10.7914/SN/ES>
- FR, RESIF. (1995). *RESIF-RLBP French Broad-band network, RESIF-RAP strong motion network and other seismic stations in metropolitan France* [Data set]. RESIF - Réseau Sismologique et géodésique Français. <https://doi.org/10.15778/RESIF.FR>
- G, Institut de physique du globe de Paris (IPGP), & École et Observatoire des Sciences de la Terre de Strasbourg (EOST). (1982). *GEOSCOPE, French Global Network of broad band seismic stations*. Institut de physique du globe de Paris (IPGP), Université de Paris. <https://doi.org/10.18715/GEOSCOPE.G>
- GB, British Geological Survey. (1970). *Great Britain Seismograph Network* [Data set]. International Federation of Digital Seismograph Networks. <https://doi.org/10.7914/av8j-nc83>
- GE, GEOFON Data Centre. (1993). *GEOFON Seismic Network* [Data set]. Deutsches GeoForschungsZentrum GFZ. <https://doi.org/10.14470/TR560404>
- GR, Federal Institute for Geosciences and Natural Resources. (1976). *German Regional Seismic Network (GRSN)*. Bundesanstalt für Geowissenschaften und Rohstoffe. <https://doi.org/10.25928/mbx6-hr74>
- GU, University of Genoa. (1967). *Regional Seismic Network of North Western Italy* [Data set]. International Federation of Digital Seismograph Networks. <https://doi.org/10.7914/SN/GU>
- HL, National Observatory of Athens, Institute of Geodynamics, Athens. (1975). *National Observatory of Athens Seismic Network* [Data set]. International Federation of Digital Seismograph Networks. <https://doi.org/10.7914/SN/HL>
- HP, University of Patras. (2000). *University of Patras, Seismological Laboratory* [Data set]. International Federation of Digital Seismograph Networks. <https://doi.org/10.7914/SN/HP>
- HU, Kövesligethy Radó Seismological Observatory (Geodetic And Geophysical Institute, Research Centre For Astronomy And Earth Sciences, Hungarian Academy Of Sciences (MTA CSFK GGI KRSZO)). (1992). *Hungarian National Seismological Network* [Data set]. GFZ Data Services. <https://doi.org/10.14470/UH028726>
- II, Scripps Institution of Oceanography. (1986). *Global Seismograph Network - IRIS/IDA* [Data set]. International Federation of Digital Seismograph Networks. <https://doi.org/10.7914/SN/II>
- IU, Albuquerque Seismological Laboratory/USGS. (2014). *Global Seismograph Network (GSN - IRIS/USGS)* [Data set]. International Federation of Digital Seismograph Networks. <https://doi.org/10.7914/SN/IU>
- IV, Istituto Nazionale di Geofisica e Vulcanologia (INGV). (2005). *Rete Sismica Nazionale (RSN)* [Data set]. Istituto Nazionale di Geofisica e Vulcanologia (INGV). <https://doi.org/10.13127/sd/x0fxnh7qfy>
- MN, MedNet Project Partner Institutions. (1990). *Mediterranean Very Broadband Seismographic Network (MedNet)* [Data set]. Istituto Nazionale di Geofisica e Vulcanologia (INGV). <https://doi.org/10.13127/sd/fbbbtdtd6q>
- NL, KNMI. (1993). *Netherlands Seismic and Acoustic Network*. Royal Netherlands Meteorological Institute (KNMI). <https://doi.org/10.21944/e970fd34-23b9-3411-b366-e4f72877d2c5>
- NO, Norsar. (1971). *NORSAR Station Network* [Data set]. NORSAR. <https://doi.org/10.21348/d.no.0001>
- NS, University of Bergen. (1982). *University of Bergen Seismic Network* [Data set]. International Federation of Digital Seismograph Networks. <https://doi.org/10.7914/SN/NS>
- OE, ZAMG - Zentralanstalt für Meterologie und Geodynamik. (1987). *Austrian Seismic Network* [Data set]. International Federation of Digital Seismograph Networks. <https://doi.org/10.7914/SN/OE>
- PM, Instituto Português do Mar e da Atmosfera, I.P. (2006). *Portuguese National Seismic Network* [Data set]. International Federation of Digital Seismograph Networks. <https://doi.org/10.7914/SN/PM>
- SL, Slovenian Environment Agency. (1990). *Seismic Network of the Republic of Slovenia* [Data set]. International Federation of Digital Seismograph Networks. <https://doi.org/10.7914/SN/SL>
- UP, Swedish National Seismic Network. (1904). *Swedish National Seismic Network* [Data set]. Uppsala University. <https://doi.org/10.18159/SNSN>

**Supplementary Figure S1**: Magnetograms (H= sqr(x^2^+y^2^) of stations at different latitude ranges during the May 2024 magnetic storm as recovered from the Intermagnet data portal (<https://intermagnet.org/new_data_download.html>).


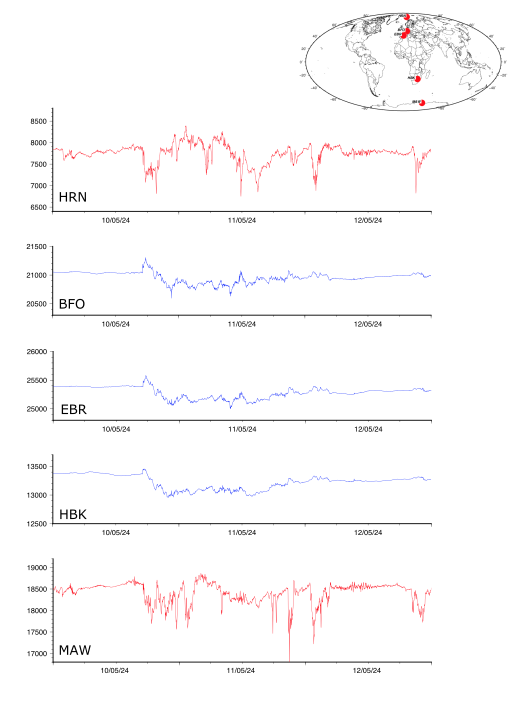


**Supplementary Figure S2:** Magnetic available in the European region at the InerMagnet facility, represented as the derivative of the horizontal magnetic field. Labels on the right show the latitude of the corresponding trace. The inset map shows, with red dots, the sites with available data.


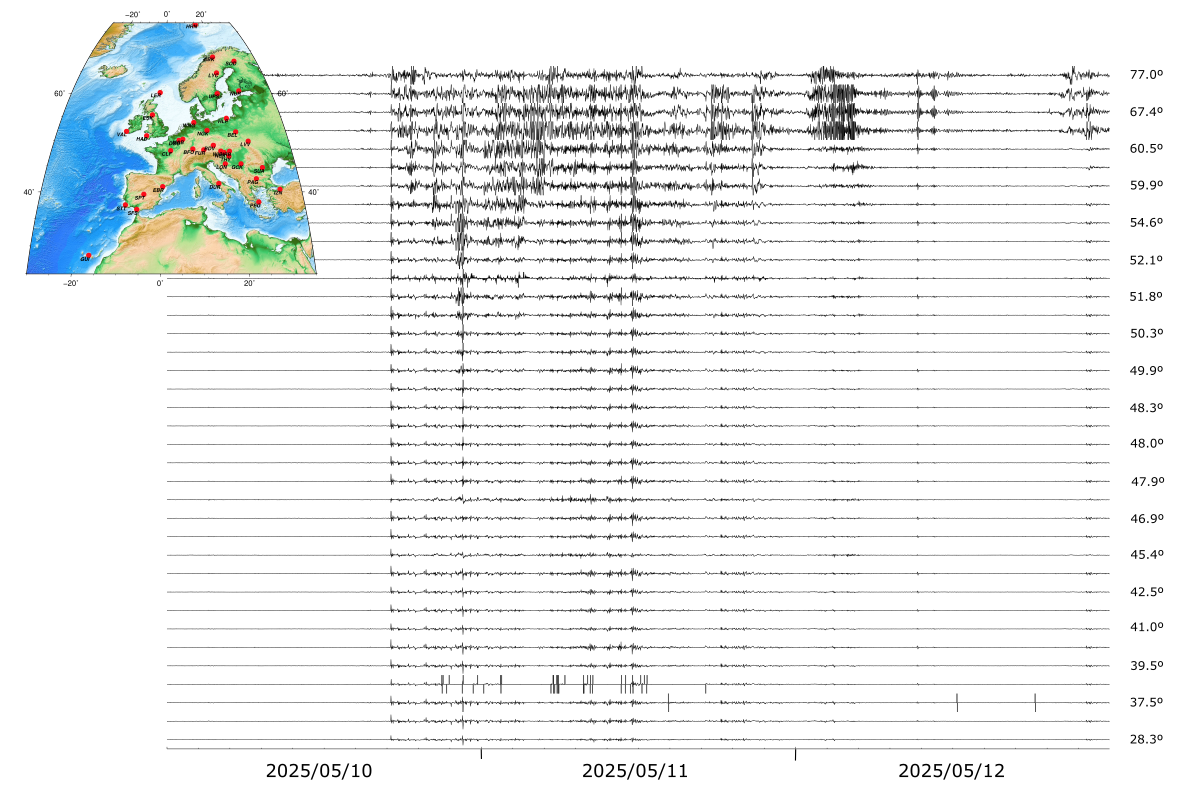


**Supplementary Figure S3:**

Seismic data acquired at station QSPA (South Pole) during the May 2024 geomagnetic storm, filtered between 1.5 and 5 mHz (a) and for the first arrivals of the Mexico 6.4 Mw earthquake, arriving the 12 May at 12:14, high-pass filtered at 10 mHz (b). Data is represented using the same amplitude scale. While for the solar storm the amplitude of the different channels differ, for the earthquake arrival all are identical, evidencing that the observed differences are not related to problems in the response files.


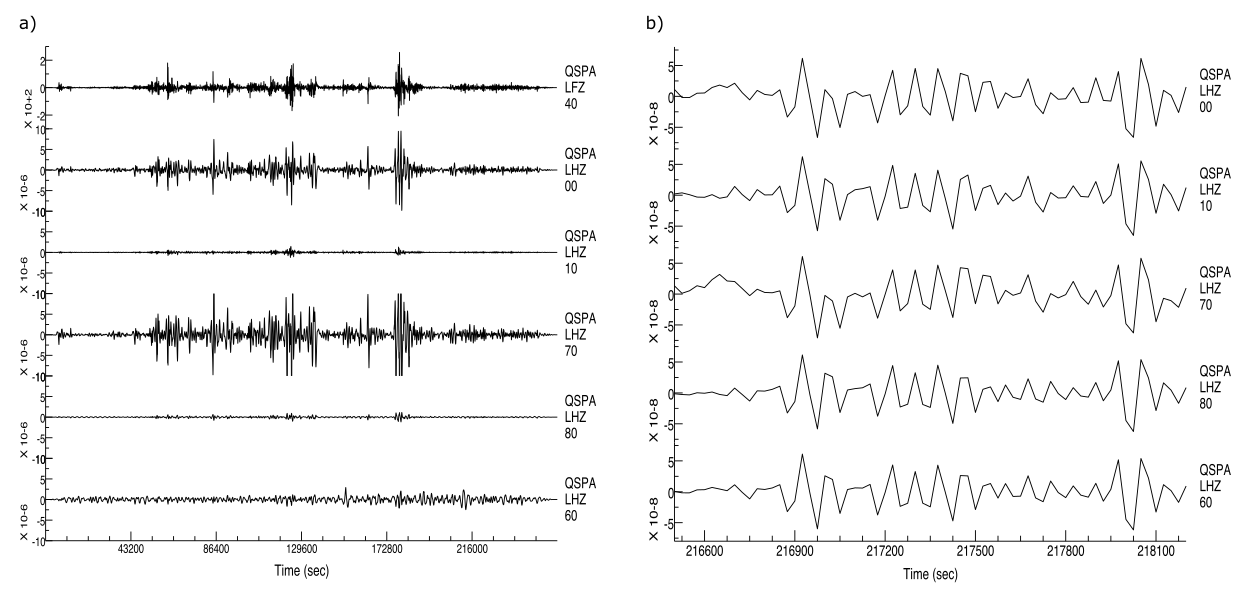


**Supplementary Figure S4:**

Seismic data acquired at the Gräfenberg array during the May 24 geomagnetic storm, filtered between 1.5 and 5 mHz (a) and first arrivals of the Mexico 6.4 Mw earthquake, arriving the 12 May at 11:39 (b). Data is represented using a normalized amplitude scale to highlight changes in polarity. Data corresponding to the seismic wave arrival show identical polarization for all the stations, while data from the solar storm show clear polarity differences.


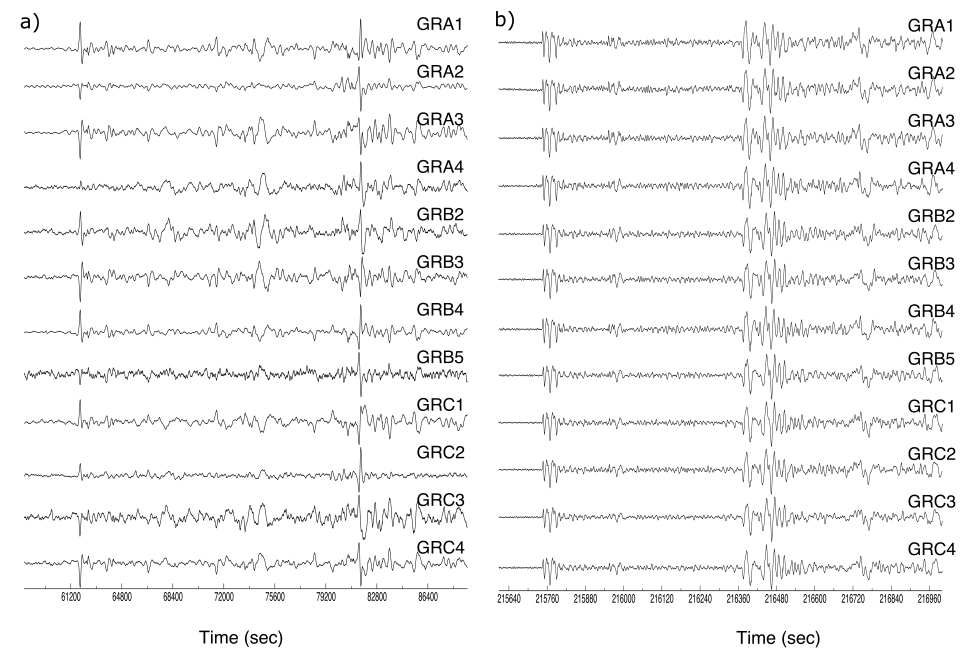

Supplement: Supplementary file 1 — Supplementary Material 1 [file 41598_2024_81079_MOESM1_ESM.docx]
